# Application of Real-Time PCR Syndromic Panel on Lower Respiratory Tract Samples: Potential Use for Antimicrobial De-Escalation

**DOI:** 10.3390/microorganisms13071678

**Published:** 2025-07-16

**Authors:** Christian Leli, Paolo Bottino, Lidia Ferrara, Luigi Di Matteo, Franca Gotta, Daria Vay, Elisa Cornaglia, Mattia Zenato, Chiara Di Bella, Elisabetta Scomparin, Cesare Bolla, Valeria Bonato, Laura Savi, Annalisa Roveta, Antonio Maconi, Andrea Rocchetti

**Affiliations:** 1 Microbiology and Virology Laboratory, University Hospital “SS Antonio e Biagio e C. Arrigo”, Via Venezia 16, 15121 Alessandria, Italy; christian.leli@ospedale.al.it (C.L.); lidia.ferrara@ospedale.al.it (L.F.); ldimatteo@ospedale.al.it (L.D.M.); fgotta@ospedale.al.it (F.G.); dvay@ospedale.al.it (D.V.); ecornaglia@ospedale.al.it (E.C.); escomparin@ospedale.al.it (E.S.); arocchetti@ospedale.al.it (A.R.); 2 Department of Science and Technological Innovation (DISIT), University of Eastern Piedmont, Viale Teresa Michel 12 11, 15121 Alessandria, Italy; mattiazenato2@gmail.com (M.Z.); chiaradibella13@gmail.com (C.D.B.); 3 Infection and Prevention Control Unit and Antimicrobial Stewardship, University Hospital “SS Antonio e Biagio e C. Arrigo”, Via Venezia 16, 15121 Alessandria, Italy; cesare.bolla@ospedale.al.it; 4 Intensive Care Unit, University Hospital “SS Antonio e Biagio e C. Arrigo”, Via Venezia 16, 15121 Alessandria, Italy; valeria.bonato@ospedale.al.it; 5 Hospital Pharmacy Management of Devices and Drugs, University Hospital “SS Antonio e Biagio e C. Arrigo”, Via Venezia 16, 15121 Alessandria, Italy; lsavi@ospedale.al.it; 6 Research and Innovation Department (DAIRI), University Hospital “SS. Antonio e Biagio e C. Arrigo”, Via Venezia 16, 15121 Alessandria, Italy; aroveta@ospedale.al.it (A.R.); amaconi@ospedale.al.it (A.M.)

**Keywords:** real-time PCR, microbiology, pneumonia, antimicrobial stewardship, diagnostics

## Abstract

Molecular methods allow for a rapid identification of the main causative agents of pneumonia along with the most frequent resistance genes. Prolonged broad-spectrum antibiotic therapy without microbiological evidence of infection drives antimicrobial resistance. We evaluated if the result provided by the molecular method is helpful for antimicrobial de-escalation. All respiratory samples collected and directly processed via Real-Time PCR from patients with suspected pneumonia, of whom clinical data were available, were included in this study. In 82 patients out of a total of 174 (47.1%), antimicrobial therapy was modified after the molecular test, and in 28/82 (34.1%), antimicrobial de-escalation was carried out. Among the 92 patients in whom therapy was not modified, 33 (35.9%) were did not receive any antimicrobial therapy before the molecular test and no antibiotics were prescribed after the test. Therefore, in 61 (28 + 33) out of the 174 (35%) patients, unnecessary antimicrobials were discontinued or avoided. The syndromic panel used at our institution can be of help in better choosing when empiric antibiotic de-escalation therapy could be feasible.

## 1. Introduction

Pneumonia is an infectious disease of the lungs involving the alveolar space and is classically divided into two broad categories, community-acquired pneumonia (CAP) and hospital-acquired pneumonia (HAP), in relation to the setting in which the disease developed [1]. Community-acquired pneumonia is mostly caused by *Streptococcus pneumoniae*; nevertheless, in a small proportion, it can also be sustained by some Gram-negative bacteria such as *Pseudomonas aeruginosa* or Enterobacterales [2]. Nosocomial pneumonia is of particular concern, in relation to the high mortality often caused by Gram-negative bacteria, sometimes carrying resistance genes [3]. It is therefore conceivable that acute pneumonia requires the optimization of an empiric antimicrobial therapy as soon as possible, as it is pivotal for the patient’s well-being [4,5,6,7].

At the same time, prolonged empiric antimicrobial therapy with broad-spectrum antibiotics without microbiological evidence of infection has been described [8,9] and is associated with an increase in bacterial antimicrobial resistance [10], mostly by ESKAPE pathogens: *Enterococcus faecium*, *Staphylococcus aureus*, *Klebsiella pneumoniae*, *Acinetobacter baumannii*, *Pseudomonas aeruginosa*, and Enterobacter species [11]. Whenever possible, antimicrobial de-escalation (ADE) should be carried out; it is the discontinuation of one or more antimicrobials or the replacement of a broad-spectrum antimicrobial with a narrower-spectrum antimicrobial [12].

Classical culture methods can take up to 48–72 h to provide an identification and an antimicrobial susceptibility result [13], and sometimes cultures are negative, particularly in patients already treated with antibiotics, even if clinical and/or radiological findings are consistent with pneumonia. Nevertheless, molecular methods applied to the diagnostics of pneumonia are becoming increasingly common [14], allowing for the rapid identification of the main bacterial/viral pathogen causative agents of pneumonia along with the most frequent resistance genes [15]. For some years now, a syndromic multiplex PCR assay has been available at our institution for the etiological diagnosis of lower respiratory tract infections.

The possible evidence of the usefulness of a rapid molecular assay as a tool for antimicrobial de-escalation would be of great help in expediting the discontinuation of unnecessary antibiotic therapies in relation to the identified microorganisms. In addition to this, the ability to quickly rule out the presence of resistance genes allows us to switch to a more targeted antibiotic therapy in the presence of bacteria susceptible to drugs already in use.

In light of the above, we conducted an observational retrospective study aimed at evaluating if the rapid identification of the pathogen and the resistance genes provided by the molecular method is helpful for antimicrobial de-escalation.

## 2. Materials and Methods

### 2.1. Study Design

This is a retrospective observational study that evaluated the period from October 2022 to July 2024. Inclusion criteria: All patients hospitalized at the Azienda Ospedaliero—Universitaria SS. Antonio e Biagio e Cesare Arrigo of Alessandria with suspected pneumonia, from whom respiratory samples were collected and directly processed by Real-Time PCR and from whom clinical data were available, were included in this study.

The criteria to define pneumonia and to differentiate CAP from HAP and ventilator-associated pneumonia (VAP) that we applied were those described by Kalil et al. [1] in the 2016 Clinical Practice Guidelines of the Infectious Diseases Society of America and the American Thoracic Society: Pneumonia is a “*new lung infiltrate plus clinical evidence that the infiltrate is of an infectious origin, which include the new onset of fever, purulent sputum, leukocytosis, and decline in oxygenation*”, defined as CAP if the infection is acquired outside of the hospital setting, but defined as HAP “*if not incubating at the time of hospital admission and occurring 48 h or more after admission*” or VAP if “*occurring >48 h after endotracheal intubation*” [1].

The criteria for ADE were the discontinuation of an antibiotic no longer supported by the microbiologic evidence or narrowed in spectrum in relation to the pathogen identified and the absence of resistance genes.

Since the interpretation of the clinical significance and the possible modification of the antimicrobial therapy were decided by the attending clinician and reported in the electronic medical record, we did not compare the molecular method’s results with those of the culture with respect to culturable pathogens or bacterial load. The clinical data were obtained from the hospital administration system TRAKCare^®^ (InterSystems, Boston, MA, USA).

### 2.2. Molecular Assay

The commercially available BioFire^®^ FilmArray^®^ Pneumonia plus Panel (BioFire Diagnostics, LLC, Salt Lake City, UT, USA) was used, according to the manufacturer’s instructions. Briefly, the pouch was rehydrated with 300 μL rehydration solution. Around 200 μL of respiratory specimen was collected with a disposable sample swab and then mixed with the provided sample buffer. The solution obtained was subsequently injected into the pouch and loaded onto the FilmArray^®^ device (Biomerieux, Marcy-l’Étoile, France) that performed amplification and detection. The Panel allows the identification of 15 bacteria (*Acinetobacter calcoaceticus baumannii* complex*, Enterobacter cloacae* complex*, Escherichia coli, Haemophilus influenzae*, *Klebsiella aerogenes*, *Klebsiella oxytoca*, *Klebsiella pneumoniae* group, *Moraxella catarrhalis*, *Proteus* spp., *Pseudomonas aeruginosa*, *Serratia marcescens*, *Staphylococcus aureus*, *Streptococcus agalactiae*, *Streptococcus pneumoniae*, and *Streptococcus pyogenes*), 3 atypical bacteria (*Chlamydophila pneumoniae*, *Legionella pneumophila*, and *Mycoplasma pneumoniae*), 8 viruses (adenovirus, coronavirus, human metapneumovirus, human rhinovirus/enterovirus, influenza A, influenza B, parainfluenza virus, and respiratory syncytial virus), and 7 resistance genes [methicillin resistance (mecA/C and mec right extremity junction: MREJ), *Klebsiella pneumoniae* carbapenemase (KPC), Verona integron-encoded metallo-Beta-lactamase (VIM), imipenemase (IMP), New Delhi metallo-β-lactamase (NDM), Oxacillinase (OXA)–48–like, and Cefotaximase-Munich (CTX-M)-type ESBL]. Results are provided within 2 h.

### 2.3. Cultural Assay

All samples were processed according to standard procedures [13]. Each sample first underwent assessment for squamous epithelial cells, inflammatory cells, and bacteria. Subsequently, it was cultured quantitatively onto Columbia agar +5% sheep blood, MacConkey agar, and *Haemophilus* Chocolate 2 agar (all media were from BioMérieux, Marcy-l’Étoile, France). Columbia agar +5% sheep blood and *Haemophilus* Chocolate 2 agar were incubated at 35 °C in 5% CO_2_, while MacConkey agar was incubated at 35 °C in ambient air. Plates were read after 24 and 48 h, and if still negative at 72 h, they were discarded. Isolates were identified using the Vitek 2^®^ system (bioMérieux, Marcy l’Etoile, France) or matrix-assisted laser desorption ionization–time of flight mass spectrometry Vitek^®^ MS (bioMérieux, Marcy l’Etoile, France).

### 2.4. Statistical Methods

Continuous variables were expressed as the median and interquartile range (IQR). Categorical variables were expressed as absolute numbers and percentages. Frequencies were compared using the McNemar Test. Microsoft Excel 2010 (Microsoft Corporation, Redmond, WA, USA) was used for the analysis. The significance level was set at *p* ≤ 0.05.

## 3. Results

A total of 174 samples from the same number of patients satisfied the inclusion criteria and were included in this study. The median age was 66 years (IQR: 54–75), and 119/174 (68.4%) were males. The requesting wards are reported in Table 1, and 163/174 (93.7%) were bronchoalveolar lavage—the others were bronchial aspirates. More than half of the samples were requested by the Intensive Care Unit (ICU), Infectious Diseases, and Pulmonology wards.

**Figure 1:**
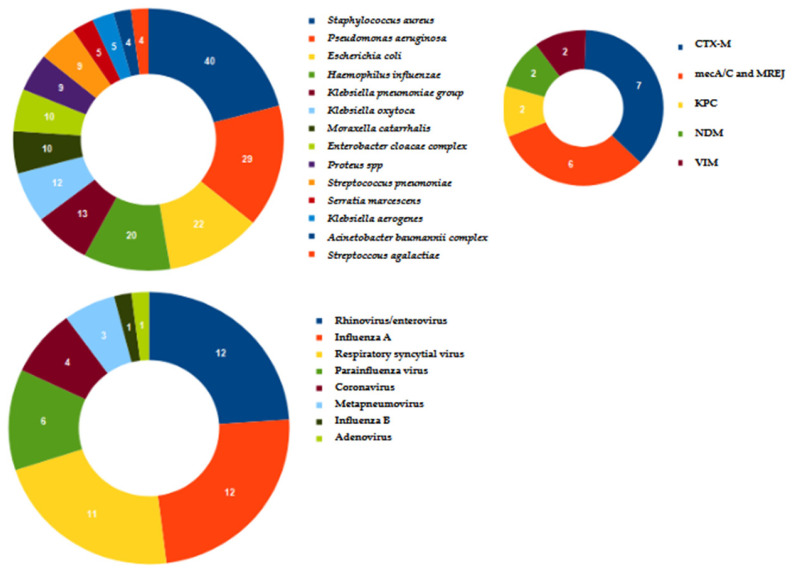
Bacteria and viruses identified using the molecular method along with the main resistance genes.

In total, 118 samples of 174 (67.8%) were positive based on the molecular assay. The microorganisms and resistance genes identified are described in Figure 1. The co-infections accounted for 63.6% (75/118) of the positive samples. Among these, 34/75 (45.3%) patients were co-infected by two pathogens, 29/75 (38.7%) by three pathogens, 11/75 (14.7%) by four, and 1/75 (1.3%) by six pathogens. The most frequent pathogen recovered in co-infection was *S. aureus* (35/75; 46.7%), followed by *P. aeruginosa* (24/75; 32%) and *E. coli* (18/75; 24%). Bacterial–viral co-infections accounted for 42.7% (32/75). Rhinovirus/enterovirus (9/32; 28.1%) and respiratory syncytial virus (8/32; 25%) were the viruses most frequently identified in bacterial–viral co-infections.

Concerning resistance genes, CTX-M and mecA/C + MREJ were the most frequent. In total, 16 samples of 118 (13.5%) were only positive for viruses.

**Figure 2:**
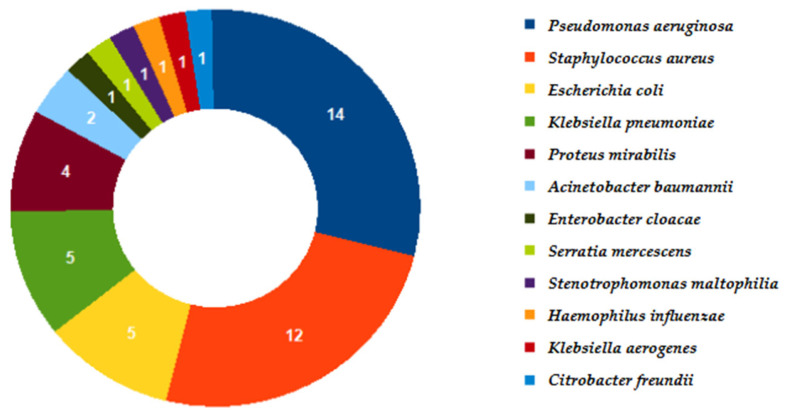
Microorganisms identified by cultural assay.

Otherwise, cultures resulted positive in 39/172 (22.7%) samples, taking into account that in two cases, culture was not requested. The microorganisms recovered are described in Figure 2. Co-infections occurred in 11/39 (28.2%) of the positive samples: 10/11 (91%) sustained by two pathogens and 1 (9%) by three pathogens. The most frequent pathogen recovered in co-infections was *S. aureus* (7/11; 63.6%), followed by *P. aeruginosa* (4/11; 36.4%).

The comparison of the two methods is reported in Table 2, and the microorganisms identified according to the proposed method are listed in Table 3. The McNemar Test yielded a significant difference (*p* < 0.0001). It is indeed evident how in 77/172 (44.8%) samples, the molecular method was positive against a negative culture. Conversely, no samples positive via culture were negative using the molecular method.

In total, 119 patients out of 174 (68.4%) were already on antimicrobial therapy before the molecular test. In 82/174 (47.1%) patients, the antimicrobial therapy was modified after the molecular test, and in 28/82 (34.1%), ADE was carried out. Among the 28 patients that underwent ADE, 12/28 (42.9%) had a positive molecular test corresponding to a negative culture and 9/28 (32%) had a negative molecular test. Therefore, 75% of ADE patients [(12 + 9)/28] had a negative culture. The remaining 7/28 (25.1%) patients underwent both a molecular test and positive culture, and the bacteria identified by Filmarray had estimates of bacterial amounts based on FA-Pneumo Bin results ranging from 10^4^ and 10^5^ DNA copies/mL. These findings from the molecular test were considered not significant by the attending physician. They corresponded to values of ≤1 × 10^3^ CFU/mL from cultures.

Conversely, in 54/82 (65.9%) patients, treatment was escalated or switched to a targeted antibiotic therapy. Among the remaining 92 patients in which therapy was not modified, 33 (35.9%) did not receive any antimicrobial therapy before the molecular test and no antibiotics were prescribed after the test. Those patients were mostly hospitalized in Intensive Care Units, often suffering from conditions such as chest injury, concurrent heart failure, hematologic malignancies, respiratory distress syndrome, high white blood cell counts, and lung attenuation on chest X-ray. In relation to the frequent hemodynamic instability and the risk of possibly worsening the clinical picture within a short time, the molecular method was used.

**Figure 3:**
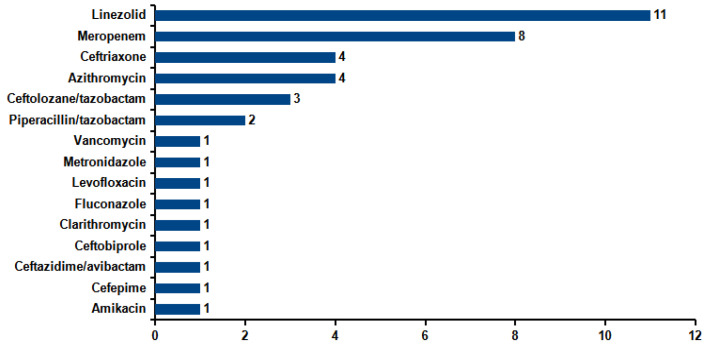
Drugs removed from empiric antimicrobial therapy according to the result of the molecular method.

Therefore, in 61 (28 + 33) out of a total of 174 (35%) patients, unnecessary antimicrobials were discontinued or avoided. Figure 3 describes the drugs removed from therapy after the result of the molecular method was available. It can be seen how the antimicrobials more frequently stopped were linezolid and meropenem. Antimicrobial de-escalation was carried out within 48–72 h of the result.

## 4. Discussion

The main result of this study is the finding of the discontinuation or avoidance of unnecessary empirical antimicrobial therapy in more than one third of patients with suspected pneumonia. Our findings substantially match those of Verroken et al. [16]. Their prospective clinical trial included 85 adult patients with severe acute pneumonia and a mean age and prevalence of male patients similar to those in this study. They found that the antimicrobial therapy was modified in about half of patients after the molecular test was performed. The proportion of those patients who underwent ADE was the same as in the present study [16]. Likewise, in the study of Buchan et al. [17] on a population of 259 adult inpatients, the authors found the potential for discontinuation or de-escalation in 48.2% of patients. Finally, similar results were also obtained by Plattner et al. [18] on a pediatric population of 126 patients, for whom a change in antibiotic therapy occurred for 46% of patients after the results of the molecular method were obtained.

Concerning the classes of discontinued antimicrobials, Stafylaki et al. [19] conducted a study including 79 patients diagnosed with pneumonia in the ICU and tested with the same panel used in this study and compared them with 40 control patients tested with conventional diagnostics. They found a significant reduction in meropenem prescription, in line with the results of the present study. Also, Monard et al. [20] conducted a retrospective multicenter study including 159 pneumonia episodes. The de-escalation of empirical antimicrobial therapy was considered in 40% of patients based on the result of the molecular method. In this study, the antibiotic suspended more frequently from empirical antibiotic therapy was linezolid. This is conceivable, since in a total of 40 S. aureus detected, only 6/40 (15%) were positive for mecA/C and MREJ.

Regarding the pathogens identified by the molecular method, in addition to the studies already described above (16–18), the results match those reported by other authors that used the same technology. Indeed, Crémet et al. [21], in a study including 100 ICU patients, found a higher prevalence of *H. influenzae*, *S. aureus*, *E. coli*, and *K. pneumoniae*. The most frequent resistance gene was CTX-M. Likewise, Mitton et al. [22] evaluated 59 lower respiratory tract specimens from subjects with suspected pneumonia, and among the most frequent pathogens identified, there were *P. aeruginosa, S. aureus*, and *K. pneumoniae* groups. In a single-center, retrospective, observational study including 163 tracheal aspirates from 109 mechanically ventilated patients, Ferrer et al. [23] reported *S. aureus* and *P. aeruginosa* as the most frequent pathogens identified. Similarly, *S. aureus*,* E. coli*, *P. aeruginosa*, and *H. influenzae* were the most frequent pathogens identified by Szymankiewicz et al. [24] in a study evaluating 79 samples of bronchoalveolar lavage. Concerning the viruses most commonly detected, the results of this study are in line with those of Webber et al. [25], who found rhinovirus/enterovirus, influenza A virus, and respiratory syncytial virus as the most frequent. Also, Van Der Westhuyzen et al. [26] identified mostly rhinovirus/enterovirus on a total of 125 lower respiratory tract samples.

With regard to the significant difference in pathogen identification between the molecular method and culture, it must be considered that nearly 70% of patients were already undergoing antimicrobial therapy at the time of testing and DNA strains of non-viable bacteria were amplified as well. Moreover, in more than 13% of the samples found positive by the molecular method, only viruses were identified, and at our laboratory, no viral culture was performed. On the other hand, culture allows us to identify *Citrobacter freundii* and *Stenotrophomonas maltophilia*, which are missed by the molecular method since it is not present in the panel. Nevertheless, those isolates were together with *S. aureus* and *K. pneumoniae*, respectively, both identified using the molecular method. Therefore, those samples are included in the set of 39 positive ones obtained using both the molecular method and culture.

The results of the present study contribute to a better understanding of the role and utility of rapid Real-Time PCR-based tests in the diagnosis of lower respiratory tract infections. On the one hand, they allow valuable information to be obtained in a short period of time that can be used to make difficult decisions for septic patients with hemodynamic instability by integrating this information with clinical history, physical examination, and radiological findings to avoid possible false positive results due to the higher sensitivity of culture. On the other hand, this study provides an experience, albeit limited to a single center, of how such tests can also be considered in antimicrobial de-escalation, an aspect so important nowadays for circumscribing the phenomenon of antibiotic resistance. Further multicenter studies with a larger sample size will allow this role to be better defined.

The effect of the rapid availability of the results of the syndromic panel can be analyzed from a pharmacoeconomic standpoint as well. Ferrer et al. [23] reported potential overall savings of EUR 6675.8 regarding antibiotic adjustment in around half of the 99 patients evaluated. In our setting, the empirical therapy for HAP involves the use of linezolid (EUR 4.08/DDD), meropenem (EUR 5.34), ceftriaxone (EUR 0.76), azithromycin (EUR 20.04), piperacillin–tazobactam (EUR 5.50), ceftolozane–tazobactam (EUR 147.06), and caspofungin (EUR 18.93), resulting in an average cost of approximately EUR 30 per defined daily dose (DDD). With the information obtained from the FilmArray Pneumonia Panel, it was possible to conduct antibiotic de-escalation in 54 out of 82 patients (65.9%) to a third-generation cephalosporin, with an average cost of EUR 1 per DDD. In considering an average antibiotic treatment duration of 10 days, the adjustment resulted in cost savings of EUR 203 per patient. Since the diagnostic test costs EUR 190, there is no direct evidence of economic benefit from antibiotic cost reduction. However, it must be considered that our evaluation was focused on susceptible bacterial strains and was not affected by antibiotic treatment for multidrug-resistant organisms. Furthermore, beyond economic considerations, the de-escalation of antibiotic therapy may offer several important clinical and public health benefits such as reduced hospital stays and faster clinical recovery, thereby improving patient outcomes, optimizing hospital resource management, and reducing indirect costs in an economy-of-scale logic [27,28].

To confirm the clinical utility of rapid molecular tests in guiding antimicrobial therapy and promoting antibiotic de-escalation in critically ill patients, a well-structured multicenter randomized controlled trial (RCT) is essential. Such a study should rely on methodological approaches already validated in the literature, including the ADAPT multicenter observational study [20] and the SCRIPT prospective cohort [29].

The proposed RCT would involve at least 10 intensive care units (ICUs) across various regions to ensure diversity in patient populations and healthcare settings. Patients with suspected hospital-acquired or ventilator-associated pneumonia (HAP/VAP) requiring empirical antibiotic therapy would be randomized 1:1 to either a standard-of-care group or an intervention group managed with rapid multiplex PCR testing on respiratory samples.

The primary endpoint would be the rate of antibiotic de-escalation at 72 h, while secondary outcomes would include total antibiotic use, ICU and hospital length of stay, the incidence of multidrug-resistant organism infections, and patient-centered outcomes such as mortality. Stratified randomization and standardized protocols for interpreting molecular results, supported by antimicrobial stewardship teams, would be vital to maintain consistency across all participating centers.

Findings from ADAPT and SCRIPT studies consistently show that rapid molecular testing significantly enhances pathogen detection compared to traditional cultures and its integration into clinical practice allows for timely de-escalation without increasing adverse outcomes. Nonetheless, single-center or retrospective designs limit the generalizability of these findings.

A multicentric study would enable broader applicability by considering regional variations in pathogen epidemiology, resistance patterns, and clinical practices. Such an approach would yield robust, high-quality evidence to guide the optimal implementation of molecular diagnostics in critical care settings globally.

The abovementioned aspect is a limitation of the present work. Furthermore, we did not have information regarding mortality, length of hospital stay, reinfection rate, and the occurrence of complications. The retrospective nature implies the possibility of selection bias, and the patients included were those for whom data were found and could be extracted. Moreover, since this is a monocentric study, the results cannot be necessarily applied to other settings. Finally, the use of a Real-Time PCR-based diagnostic tool, on one hand, requires expensive disposables and equipment and qualified people; on the other hand, it has the risk of reporting a positive a sample that potentially contains only DNA from microorganisms already killed via antibiotic therapy. Defining a meaningful threshold of DNA copies/mL is the subject of an ongoing study.

## 5. Conclusions

This study showed how the syndromic panel in use at our institution not only helps carry out a faster etiological diagnosis of pneumonia but also allows us to better choose when empiric antibiotic therapy de-escalation could be feasible.

## Figures and Tables

**Table 1:** Details of wards involved in the study.

Ward	N	%
Intensive Care	61	35.1
Infectious Diseases	20	11.5
Pulmonology	28	16.1
Hematology	13	7.5
Sub-Intensive Care	13	7.5
Pediatric Intensive Care	10	5.7
Emergency Medicine	8	4.5
Internal Medicine	5	2.9
Coronary Care Unit	3	1.7
General Surgery	2	1.1
Pediatrics	2	1.1
Oncology	2	1.1
Gastroenterology	1	0.6
Nephrology	1	0.6
Neurology	1	0.6
Pediatric Emergency Room	1	0.6
Respiratory Rehabilitation	1	0.6
Spinal Unit	1	0.6
Urology	1	0.6
Total	174	100

**Table 2:** Comparison of the molecular and cultural methods.

		Cultural Method		
		Positive	Negative	Total
Molecular assay	Positive	39	77	116
	Negative	0	56	56
	Total	39	133	172

**Table 3:** Microorganisms identified according to proposed method.

Microorganism	Identified only by Molecular Methods	Identified only by Culture	Identified by Both Methods
*Acinetobacter baumannii*	2	0	2
*Klebsiella pneumoniae*	8	0	5
*Pseudomonas aeruginosa*	15	0	14
*Proteus* spp.	5	0	4
*Enterobacter cloacae complex*	9	0	1
*Escherichia coli*	17	0	5
*Haemophilus influenzae*	19	0	1
*Staphylococcus aureus*	28	0	12
*Stenotrophomonas maltophilia*	0	1	0
*Klebsiella oxytoca*	12	0	0
*Citrobacter freundii*	0	1	0
*Streptococcus agalactiae*	4	0	0
*Moraxella catarrhalis*	10	0	0
*Streptococcus pneumoniae*	9	0	0
*Klebsiella aerogenes*	4	0	1
*Serratia marcescens*	4	0	1
Total	146	2	46

## Data Availability

The original contributions presented in this study are included in the article. Further inquiries can be directed to the corresponding author.
